# Generative AI acceptance and professional autonomy development among Chinese university physical education teachers: a moderated conditional process model

**DOI:** 10.3389/fpsyg.2026.1884187

**Published:** 2026-07-21

**Authors:** Xiao Chen, Qishun Yang, Mingliang Song, Yuejun Zheng, Chen Lei

**Affiliations:** 1 School of Football, Wuhan Sports University, Wuhan, China; 2 School of Physical Education and International Equestrian College, Wuhan Business University, Wuhan, Hubei, China; 3 School of Sports Training, Tianjin University of Sport, Tianjin, China; 4 Department of Physical Education, Zhongnan University of Economics and Law, Wuhan, Hubei, China

**Keywords:** generative artificial intelligence, learning motivation, teacher professional autonomy development, teaching innovation, technology anxiety, university physical education teachers

## Abstract

**Objective:**

Generative artificial intelligence (GenAI) is increasingly being integrated into educational settings, reshaping teaching practices and teacher professional development. This study examined whether GenAI acceptance was associated with teacher professional autonomy development (PAD) among university physical education teachers through the indirect roles of teaching innovation and learning motivation, and whether technology anxiety moderated these associations.

**Methods:**

The study included a preliminary pilot survey and a main questionnaire survey. The pilot survey assessed item clarity, contextual suitability, feasibility, and preliminary reliability. Formal hypothesis testing was based on main-survey data from 1,020 Chinese university physical education teachers. A moderated serial conditional process model was estimated using PROCESS Model 92 with observed composite scores and 5,000 bootstrap resamples.

**Results:**

GenAI acceptance was significantly associated with PAD (total effect = 0.563, 95% CI [0.512, 0.615]). Teaching innovation and learning motivation showed significant indirect associations, accounting for 20.07% and 15.81% of the total association, respectively. The serial indirect association involving both teaching innovation and learning motivation accounted for an additional 5.15% (effect = 0.029, 95% CI [0.018, 0.041]). Technology anxiety weakened the positive associations between GenAI acceptance and both teaching innovation and learning motivation, with stronger attenuation observed for the teaching innovation association.

**Conclusions:**

The findings suggest that GenAI acceptance is positively associated with PAD among university physical education teachers and that teaching innovation and learning motivation may represent indirect correlates of this association. Technology anxiety may weaken these associations. Because teaching innovation, learning motivation, and PAD were measured in the same main survey, the findings should be interpreted as regression-based conditional process associations rather than causal, cross-lagged, or developmental effects.

## Introduction

Teacher professional autonomy development (PAD) refers to the dynamic process through which teachers, guided by personal needs and professional goals, enhance their professional competence through proactive learning and reflective practice (Çolak, 2024). China's Education Modernization 2035 emphasizes educational reform in the information age and highlights the importance of teacher professional autonomy development for talent cultivation in the new era (Zhu, 2019). University physical education teachers are central to promoting students‘ physical and mental health, improving the quality of physical education instruction, and supporting students' physical fitness outcomes (Kim and Cruz, 2024). However, recent evidence indicates a decline in physical fitness indicators among Chinese university students. The prevalence of overweight/obesity, elevated blood pressure, and their comorbidity among Chinese college students increased from 3.7%, 2.2%, and 0.3% in 2000 to 14.0%, 5.2%, and 1.8% in 2019, respectively (Cai et al., 2025). These trends highlight limitations in traditional physical education classes, especially in personalized guidance and scientific monitoring, and point to the need for physical education teachers to update their knowledge and professional competencies (Hu and Min, 2025). With the rapid development of generative artificial intelligence (GenAI) in education, technology has moved beyond content storage and course management toward platforms that support content generation, real-time feedback, and instructional decision-making (Johri et al., 2023). This transformation creates new opportunities for teacher development, particularly in practice-intensive and feedback-dependent fields such as physical education.

GenAI may support evidence-informed training recommendations by drawing on large-scale sports databases and the exercise science literature, enabling teachers to supplement personal experience with broader data-informed pedagogical resources (Pietraszewski et al., 2025). Studies on AI-assisted coaching systems also indicate that incorporating data analytics into instruction can be associated with stronger professional confidence and closer alignment between teaching practices and contemporary sports science principles (Claudino et al., 2019). In this sense, GenAI may optimize instructional workflows and reshape teachers‘ professional development pathways. Yet the process through which the technological affordances of GenAI are translated into physical education teachers' PAD remains underexplored.

Although prior studies have examined the educational potential of GenAI, much of this work has focused on direct outcomes, such as teaching ability or teaching efficacy (MacDowell et al., 2024). Less is known about the indirect mechanisms through which GenAI acceptance is associated with professional growth through teachers‘ behavioral and psychological processes. Empowerment theory proposes that external resources can enhance individuals' sense of control and competence and thereby activate proactive behavioral responses (Thomas and Velthouse, 1990). Within this framework, teaching innovation refers to teachers‘ systematic transformation of traditional teaching models to enhance students' comprehensive competencies (Sofi-Karim et al., 2023). It can therefore be understood as a behavioral mechanism through which external technological resources are linked to professional development outcomes. In GenAI-enabled physical education, teaching innovation involves instructional redesign, embodied demonstration strategies, and adaptive coaching in dynamic environments where motor skill assessment and injury prevention require timely decision-making (Baek et al., 2018). Empirical evidence suggests that GenAI acceptance is positively associated with teachers‘ restructuring of instructional frameworks and willingness to engage in innovative teaching practices (Scott and Smith, 2024). Teaching innovation may therefore represent an indirect correlate in the association between GenAI acceptance and teachers' PAD.

Whether external technological support is translated into sustained professional development also depends on teachers‘ motivational appraisal of the technology context. Self-determination theory proposes that autonomy, competence, and relatedness support autonomous and internalized forms of learning motivation and sustained investment in learning (Deci and Ryan, 2000). Learning motivation refers to the behavioral drive shaped by perceived goal value and self-efficacy within specific instructional contexts, and it influences teachers' engagement in professional learning tasks (Bandura, 1978). In GenAI-enabled classrooms, learning motivation may serve as a psychological link between technology adoption and teacher professional growth. Studies have shown that teachers‘ learning motivation increases after GenAI training and resource support, providing a motivational basis for sustained technology use (Verano-Tacoronte et al., 2025). In physical education, teachers whose motivation is grounded in curiosity about sports science innovation and professional development goals may be more likely to participate in AI-related professional development and to integrate data-driven pedagogies into teaching practice (Banville and Rikard, 2009). Integrating empowerment theory and self-determination theory, this study conceptualizes teaching innovation and learning motivation as interconnected indirect correlates of teachers' professional development.

Teachers‘ emotional attitudes and cognitive evaluations of technological tools also shape the effectiveness of technological empowerment (Dignath et al., 2022). Technology anxiety, defined as the negative emotions and psychological burden experienced when using new technologies, has been repeatedly identified as a barrier to teachers' technology adoption (Henderson and Corry, 2021) and behavioral engagement (Falebita, 2025). Because physical education teaching often occurs in open, dynamic, and risk-sensitive environments, technology anxiety among physical education teachers may be especially situation-sensitive and may function as a boundary condition for the relationship between GenAI acceptance and PAD. Existing empirical work has focused mainly on teachers in non-physical education disciplines, and much of the available evidence has been derived from Western teacher populations (Han, 2025). Evidence from Asian higher education contexts, particularly among university physical education teachers, remains limited.

To address these gaps, the present study focuses on Chinese university physical education teachers and examines a theoretically grounded conditional process model in which GenAI acceptance is associated with PAD through teaching innovation and learning motivation, with technology anxiety as a moderator. Because the present design does not establish temporal ordering among teaching innovation, learning motivation, and PAD, the proposed model is interpreted as a set of conditional process associations rather than as evidence of causal or developmental sequencing.

H1: GenAI acceptance is positively associated with university physical education teachers' PAD.

H2: Teaching innovation shows an indirect role in the association between GenAI acceptance and PAD.

H3: Learning motivation shows an indirect role in the association between GenAI acceptance and PAD.

H4: Teaching innovation and learning motivation jointly form a serial indirect association between GenAI acceptance and PAD.

H5: Technology anxiety negatively moderates the association between GenAI acceptance and teaching innovation among university physical education teachers.

**Figure 1:**
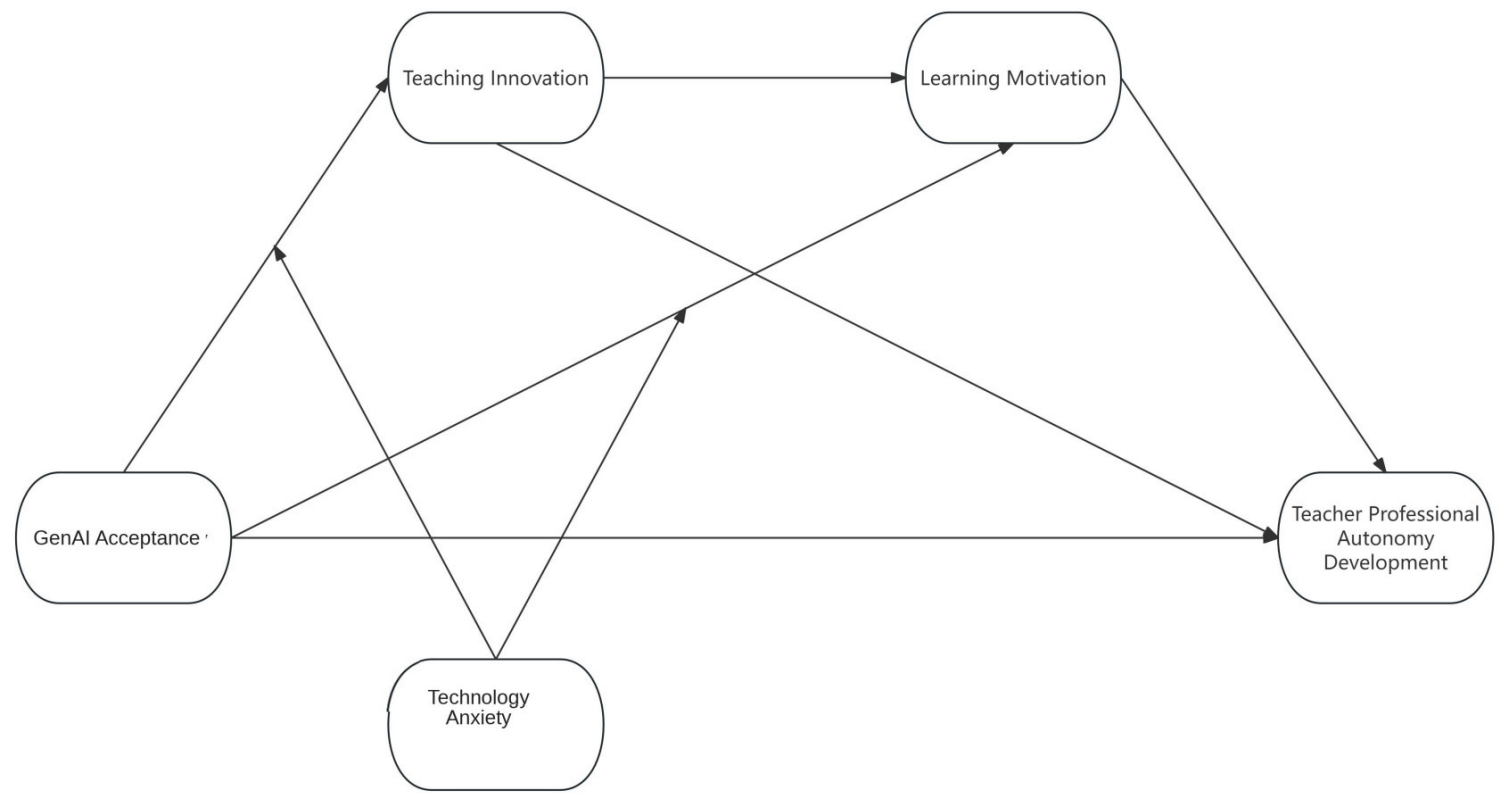
Hypothesized model.

H6: Technology anxiety negatively moderates the association between GenAI acceptance and learning motivation among university physical education teachers (Figure 1).

## Materials and methods

### Participants

This study adopted a questionnaire-based research design consisting of a preliminary pilot survey and a main questionnaire survey among university physical education teachers in China. The preliminary pilot survey was conducted from March 5 to March 15, 2025, and was used to examine the clarity, feasibility, contextual suitability, and preliminary reliability of the questionnaire items. Based on the pilot feedback, minor wording adjustments were made to ensure that the adapted instruments were appropriate for the professional context of university physical education teachers. The pilot survey data were used only for questionnaire refinement and were not included in the formal hypothesis testing.

The main questionnaire survey was conducted from June 20 to June 30, 2025. All participants in the main survey completed measures of GenAI acceptance, teaching innovation, learning motivation, PAD, and technology anxiety. To reduce the risk of common method bias, the questionnaire was administered anonymously, participants were assured that there were no right or wrong answers, and all responses were used only for academic research purposes. Responses were anonymized using numeric coding.

A stratified sampling strategy (Cochran, 1946) was used to improve geographic coverage across China's 31 provincial-level administrative regions. Sampling was first organized by regional strata; sample quotas were then allocated proportionally according to the number of universities within each stratum, followed by the selection of institutions within each region. Full-time physical education faculty members in the selected universities received the questionnaire link. After incomplete responses and patterned answers were excluded, 1,020 valid questionnaires were retained from 1,228 initial respondents, yielding an effective response rate of 83.1%. Demographic characteristics of the sample are presented in Table 1. Participants who completed the main questionnaire received a small token of appreciation.

**Table 1:** Demographic characteristics.

Variables		*N* (%)
Gender	Male	711 (69.7%)
	Female	309 (30.3%)
Marital status	Married	726 (71.2%)
	Unmarried	294 (28.8%)
Age	18–25	127 (12.5%)
	26–30	221 (21.7%)
	31–40	238 (23.3%)
	41–50	259 (25.4%)
	51–60	139 (13.6%)
	Above 60	36 (3.5%)
Work regions	Central China	148 (14.5%)
	East China	184 (18%)
	South China	165 (16.2%)
	North China	123 (12.1%)
	Southwest	160 (15.7%)
	Northwest	106 (10.4%)
	Northeast China	134 (13.1%)
Professional titles	Assistant lecturer	119 (11.7%)
	Lecturer	734 (72%)
	Associate professor	131 (12.8%)
	Full professor	36 (3.5%)
Educational backgrounds	Bachelor	164 (16.1%)
	Master	621 (60.9%)
	Doctor	235 (23%)
Teaching Experience	Less than 5 years	294 (28.8%)
	5–10 years	312 (30.6%)
	11–20 years	281 (27.5%)
	More than 20 years	133 (13.1%)

### Ethics statement

All procedures involving human participants were conducted in accordance with the ethical standards of the institutional research committee and with the 1964 Declaration of Helsinki and its later amendments. The study protocol was reviewed and approved by the Academic Ethics Committee of Tianjin University of Sport (Ethical Approval No. TJUS2025-079).

### Informed consent

Electronic informed consent was obtained from all participants before data collection. The first page of the online questionnaire explained the study objectives, voluntary participation, anonymity procedures, data use, and the right to withdraw at any time without penalty. Only participants who actively indicated consent were allowed to proceed to the survey.

### Instruments

All English-language instruments were adapted into Chinese following standardized cross-cultural adaptation guidelines (Beaton et al., 2000). The procedure consisted of three steps: (1) forward translation by two independent bilingual researchers with expertise in educational psychology and physical education; (2) synthesis and reconciliation by a three-member expert panel to resolve discrepancies and ensure semantic equivalence; and (3) back-translation by an independent translator who was unfamiliar with the original scales to verify conceptual fidelity.

#### Generative artificial intelligence

The Generative Artificial Intelligence Acceptance Scale was developed by Yilmaz et al. (2024) to systematically assess individuals' acceptance and perceived usefulness of generative AI applications. The scale comprises 20 items covering four dimensions: Performance Expectancy (e.g., “I find generative AI applications useful in my daily life”), Effort Expectancy (e.g., “Learning how to use generative AI applications is easy for me”), Facilitating Conditions (e.g., “Generative AI applications are compatible with other technologies I use”), and Social Influence (e.g., “People important to me think I should use generative AI applications”). All items are rated on a five-point Likert scale (1 = strongly disagree, 5 = strongly agree). Reliability and validity tests showed excellent psychometric properties: the overall scale demonstrated a Cronbach's α of 0.935, with subscale α coefficients ranging from 0.859 to 0.912. Confirmatory factor analysis (CFA) indicated good model fit (χ^2^/df = 1.145, RMSEA = 0.012, SRMR = 0.016, IFI = 0.998, TLI = 0.998, CFI = 0.998, GFI = 0.943). Both the average variance extracted (AVE = 0.5973–0.6074) and composite reliability (CR = 0.8593–0.9121) exceeded psychometric thresholds (Joseph et al., 2018), indicating adequate measurement reliability and structural validity.

#### Teacher professional autonomy development

Teacher professional autonomy development was measured using the Teacher Autonomous Behavior Scale developed by Evers et al. (2017). The original scale assesses teacher autonomy across four dimensions: primary work processes in the classroom, curriculum implementation, participation in school decision-making, and professional development, with 24 items in total. These dimensions are considered core elements of teacher professional autonomy. To ensure a consistent response format across the questionnaire and reduce respondent burden, the original seven-point Likert response format was rescaled to a five-point format, ranging from 1 = strongly disagree to 5 = strongly agree.

The psychometric refinement process followed both statistical and theoretical criteria. We first conducted a confirmatory factor analysis on the full 24-item version. Four items were removed because their standardized factor loadings were below 0.60. Specifically, the deleted items were TAB4: “autonomy in arranging classroom activities” from the primary work processes dimension, TAB10: “autonomy in adjusting prescribed curriculum content” from the curriculum implementation dimension, TAB16: “influence on school-level policy decisions” from the participation in school decision-making dimension, and TAB22: “autonomy in choosing professional development activities” from the professional development dimension. Their standardized factor loadings were 0.548, 0.563, 0.521, and 0.574, respectively.

The deletion of these items did not remove any theoretical dimension from the scale. The final 20-item version retained five items in each of the four original dimensions, thereby preserving the conceptual structure of the original instrument. The retained items showed satisfactory standardized factor loadings, ranging from 0.671 to 0.842. Reliability and validity analyses of the final 20-item scale demonstrated satisfactory psychometric performance. The overall scale exhibited a Cronbach's α of 0.935, with subscale α coefficients ranging from 0.844 to 0.902. The CFA model fit indices for the refined scale were satisfactory: χ^2^/df = 1.199, RMSEA = 0.014, SRMR = 0.018, IFI = 0.997, TLI = 0.997, CFI = 0.997, and GFI = 0.981. The AVE values ranged from 0.575 to 0.6129, and the CR values ranged from 0.8439 to 0.9048. These results provide evidence of internal consistency and factorial validity of the adapted scale in the present sample.

#### Teaching innovation

The measurement of teaching innovation employed the Teacher's Innovative Work Behavior scale developed by Zhang and Zhang (2012). This scale includes 16 items across three core dimensions: innovation idea (e.g., “I proactively learn from others' teaching experiences”), innovation action (e.g., “I flexibly apply different teaching methods to impart knowledge and skills”), and innovation result (e.g., “I encourage students to think about and discuss controversial issues”). All items are scored on a five-point Likert scale (1 = strongly disagree, 5 = strongly agree). Reliability tests showed a Cronbach's α of 0.924 for the overall scale, with subscale α coefficients ranging from 0.880 to 0.915. Confirmatory factor analysis (CFA) demonstrated good model fit: χ^2^/df = 1.287, RMSEA = 0.017 (90% CI: 0.013–0.021), SRMR = 0.017, IFI = 0.996, TLI = 0.997, CFI = 0.997, and GFI = 0.984. Both the average variance extracted (AVE = 0.572–0.642) and composite reliability (CR = 0.870–0.915) met established thresholds (AVE > 0.50, CR > 0.70), indicating adequate measurement reliability and construct validity.

#### Learning motivation

The learning motivation scale was adapted from the Questionnaire on Learning Motivation of Distance Learners developed by Wang et al. (2008) and revised for university physical education teachers. Because the original instrument was developed for distance-learning contexts, contextual wording adaptation was necessary. Terms such as “distance learning” were replaced with “GenAI-assisted professional learning” or “adoption of emerging educational technologies.” This adaptation was intended to capture motivational drivers in a technology-enhanced sports education context.

No item was deleted from the learning motivation scale. All 33 adapted items corresponding to the original instrument were retained. The adapted scale covered three dimensions: cognitive learning motivation, career-development motivation, and social-recognition motivation. These labels were used to reflect the substantive content of the adapted items and to avoid treating career- or recognition-oriented motives as purely intrinsic motivation in the strict sense of self-determination theory.

All items were measured on a five-point Likert scale ranging from 1 to 5. Empirical tests conducted on the target sample of university physical education teachers indicated high internal consistency, with an overall Cronbach's α of 0.965 and subscale reliabilities ranging from 0.944 to 0.948. Confirmatory factor analysis demonstrated excellent model fit: χ^2^/df = 1.119, RMSEA = 0.011, SRMR = 0.017, CFI = 0.998, and TLI = 0.997. The AVE values ranged from 0.603 to 0.626, and the CR values ranged from 0.944 to 0.949. These results support the reliability and factorial validity of the adapted learning motivation scale among Chinese university physical education teachers.

#### Technology anxiety

Technology anxiety was measured using the Information Technology Anxiety Scale (ITAS) developed by López-Bonilla and López-Bonilla (2012). This scale, grounded in perceived barriers theory within the Technology Acceptance Model, consists of 10 unidimensional items (e.g., “I feel apprehensive about using new technologies”). All items are rated on a 5-point Likert scale (1 = strongly disagree, 5 = strongly agree). Psychometric evaluation demonstrated excellent internal consistency (Cronbach's alpha = 0.941). To verify the scale's factor structure in the faculty population, exploratory factor analysis (EFA) was performed. The Kaiser-Meyer-Olkin measure of sampling adequacy was 0.970, Bartlett's test of sphericity was significant (chi-square = 6799.414, *p* < 0.001), and a single factor with an eigenvalue greater than 1 explained 65.16% of the total variance. Standardized factor loadings ranged from 0.767 to 0.805, supporting the reliability and structural validity of the scale for measuring technology anxiety among university teachers.

#### Statistical analysis

After data collection, SPSS 27.0 and Amos 28.0 were used for data analysis. To assess construct validity, a combined five-factor measurement model including GenAI acceptance, teaching innovation, learning motivation, PAD, and technology anxiety was estimated simultaneously using confirmatory factor analysis (CFA). All latent constructs were allowed to correlate freely. The model fit supported the distinctiveness of the five constructs. Normality tests showed that the absolute values of skewness for all variables were within 2 and kurtosis values were within 7 (Kline, 2016), suggesting approximate normality. Descriptive statistics were calculated, and demographic differences were examined using analysis of variance (ANOVA) and independent samples t-tests. Pearson's r correlation coefficients were computed to examine linear relationships among variables, with effect size interpretations based on Cohen's (1988) guidelines: 0.1 to 0.3 indicating small effects, 0.3 to 0.5 medium effects, and above 0.5 large effects.

Following Hayes (2018), a moderated serial conditional process model was tested using Model 92 of the PROCESS macro (version 4.2) in SPSS (Hayes, 2022). The model examined indirect associations through teaching innovation and learning motivation between GenAI acceptance and PAD, as well as the moderating role of technology anxiety. Because the indirect variables were measured in the same main survey, the estimated indirect associations were interpreted as regression-based statistical associations rather than causal mediation effects or evidence of temporal sequencing. Bias-corrected 95% confidence intervals were estimated using 5,000 bootstrap resamples (Preacher and Hayes, 2008; Shrout and Bolger, 2002). Mediation and moderation analyses were conducted using observed composite scores rather than latent constructs, which facilitates interpretation but does not explicitly model measurement error.

## Results

### Common method bias and discriminant validity

Data quality was first assessed using Harman's single-factor test (Podsakoff et al., 2003). Exploratory factor analysis extracted 15 factors with eigenvalues greater than 1, explaining 67.3% of the total variance. The first factor accounted for 29.3% of the total variance, below the commonly referenced 40% threshold.

Subsequently, competitive confirmatory factor models were constructed (see Table 2). The single-factor model exhibited poor fit (χ^2^/df = 15.698, RMSEA = 0.120, CFI = 0.758), whereas the five-factor theoretical model demonstrated good fit (χ^2^/df = 1.083, RMSEA = 0.009, CFI = 0.999), supporting the multidimensional structure of the constructs.

**Table 2:** Common method bias test.

Model	CMIN/DF	RMSEA	SRMR	IFI	CFI	GFI
Single-factor model	15.698	0.120	0.0788	0.758	0.758	0.793
Five-factor model (theoretical)	1.083	0.009	0.0168	0.999	0.999	0.987
Five-factor model + ULMC	1.062	0.008	0.017	0.999	0.999	0.987

ULMC, Unmeasured latent method construct.

Finally, the unmeasured latent method construct (ULMC) approach was applied by introducing a common method factor into the theoretical model. Inclusion of the ULMC resulted in negligible changes in model fit indices (Delta CFI = 0.000, Delta RMSEA = −0.001, Delta SRMR = 0.0002).

Taken together, these findings suggest that common method bias is unlikely to substantially distort the primary structural relationships (Williams and McGonagle, 2016); however, residual method variance cannot be entirely ruled out.

To further examine construct distinctiveness, discriminant validity was assessed using the Heterotrait–Monotrait (HTMT) ratio. HTMT ratios were computed for all construct pairs (see Table 3). Values ranged from 0.475 to 0.693, all below the conservative threshold of 0.85 (Henseler et al., 2015), indicating satisfactory discriminant validity.

**Table 3:** Heterotrait–Monotrait ratios.

Variable	GenAI	TI	LM	PAD	TA
GenAI	—				
TI	0.682	—			
LM	0.599	0.624	—		
PAD	0.693	0.627	0.627	—	
TA	0.483	0.497	0.475	0.510	—

GenAI, generative artificial intelligence; TI, teaching innovation; LM, learning motivation; PAD, professional autonomy development; TA, technology anxiety.

Collectively, these findings provide consistent evidence that the five constructs are empirically distinguishable.

### Measurement invariance and subgroup reliability

To examine whether the revised measures functioned comparably across different teacher groups, multigroup confirmatory factor analyses were conducted for the two most extensively adapted measures: the Teacher Autonomous Behavior Scale and the learning motivation scale. Measurement invariance was examined across gender groups (male vs. female) and teaching-experience groups (less than 5 years, 5–10 years, 11–20 years, and more than 20 years). Configural, metric, and scalar invariance were evaluated using changes in CFI, RMSEA, and SRMR (Vandenberg and Lance, 2000).

For the Teacher Autonomous Behavior Scale, the gender-group invariance tests supported configural invariance (CFI = 0.995, RMSEA = 0.015, SRMR = 0.021). Imposing equality constraints on factor loadings produced only negligible changes in model fit (ΔCFI = −0.001, ΔRMSEA = 0.001, ΔSRMR = 0.003), supporting metric invariance. Further constraining item intercepts also produced acceptable changes in model fit (ΔCFI = −0.001, ΔRMSEA = 0.000, ΔSRMR = 0.004), supporting scalar invariance. The teaching-experience-group tests showed a similar pattern, supporting configural invariance (CFI = 0.993, RMSEA = 0.017, SRMR = 0.026), metric invariance (ΔCFI = −0.001, ΔRMSEA = 0.000, ΔSRMR = 0.004), and scalar invariance (ΔCFI = −0.001, ΔRMSEA = 0.001, ΔSRMR = 0.005).

For the learning motivation scale, the gender-group tests supported configural invariance (CFI = 0.996, RMSEA = 0.013, SRMR = 0.020), metric invariance (ΔCFI = −0.001, ΔRMSEA = 0.000, ΔSRMR = 0.003), and scalar invariance (ΔCFI = −0.001, ΔRMSEA = 0.001, ΔSRMR = 0.004). The teaching-experience-group tests also supported configural invariance (CFI = 0.994, RMSEA = 0.015, SRMR = 0.024), metric invariance (ΔCFI = −0.001, ΔRMSEA = 0.001, ΔSRMR = 0.004), and scalar invariance (ΔCFI = −0.001, ΔRMSEA = 0.001, ΔSRMR = 0.005). These results suggest that the adapted measures showed acceptable measurement invariance across gender and teaching-experience groups.

Subgroup-specific reliability coefficients also supported measurement stability. The Teacher Autonomous Behavior Scale showed stable internal consistency across gender groups (α = 0.936 for male teachers; α = 0.931 for female teachers) and teaching-experience groups (α = 0.928–0.939). The learning motivation scale also showed stable reliability across gender groups (α = 0.966 for male teachers; α = 0.962 for female teachers) and teaching-experience groups (α = 0.961–0.968). Together, these findings provide additional evidence that the revised measures functioned consistently across the teacher subgroups examined.

### Correlation analysis

Table 4 presents Pearson correlation coefficients among the key variables. GenAI acceptance showed a strong positive correlation with PAD (r = 0.560, *p* < 0.01) and learning motivation (r = 0.564, *p* < 0.01), and a moderate positive correlation with teaching innovation (r = 0.477, *p* < 0.01). These results support the hypothesis that GenAI acceptance is positively related to PAD among university physical education teachers and provide a statistical basis for the subsequent conditional process analysis. Technology anxiety was moderately negatively correlated with GenAI acceptance (r = −0.425, *p* < 0.01), teaching innovation (r = −0.405, *p* < 0.01), and learning motivation (r = −0.444, *p* < 0.01), consistent with its role as a negative correlate and candidate boundary condition.

**Table 4:** Correlation analysis.

Variable	Mean ±SD	GenAI	TI	LM	PAD	TA
GenAI	3.498 ± 0.786	–				
TI	3.483 ± 0.804	0.477^**^	–			
LM	3.46 ± 0.814	0.564^**^	0.495^**^	–		
PAD	3.448 ± 0.79	0.56^**^	0.496^**^	0.511^**^	–	
TA	2.617 ± 0.939	−0.425^**^	−0.405^**^	−0.444^**^	−0.437^**^	–

^**^p < 0.01, GenAI, generative artificial intelligence; TI, teaching innovation; LM, learning motivation; PAD, professional autonomy development; TA, technology anxiety.

### Indirect association model testing

**Figure 2:**
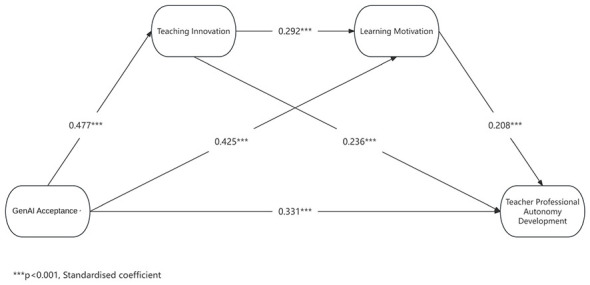
Indirect association model.

The conditional process model showed that GenAI acceptance had a significant total association with PAD (effect = 0.563, 95% CI [0.512, 0.615]). The direct association was 0.332 (95% CI [0.272, 0.392]), accounting for 58.97% of the total association. The total indirect association accounted for 41.03% of the total association (effect = 0.231) and was transmitted through three indirect paths. The strongest independent indirect association was through teaching innovation (effect = 0.113, 95% CI [0.084, 0.146], 20.07%), followed by learning motivation (effect = 0.089, 95% CI [0.059, 0.122], 15.81%). The serial indirect association through teaching innovation and learning motivation contributed an additional association (effect = 0.029, 95% CI [0.018, 0.041], 5.15%) (Table 5, Figure 2).

**Table 5:** Indirect association effect sizes.

Effect type or pathway	Effect	SE	95% confidence interval		Proportion of Total effect
			LLCI	ULCI	
Total effect	0.563	0.026	0.512	0.615	
Direct effect	0.332	0.031	0.272	0.392	58.97%
Total indirect effect	0.231	0.023	0.189	0.279	41.03%
GenAI ->TI-> PAD	0.113	0.016	0.084	0.146	20.07%
GenAI ->LM-> PAD	0.089	0.016	0.059	0.122	15.81%
GenAI ->TI-> LM ->PAD	0.029	0.006	0.018	0.041	5.15%

The confidence interval of the pathway did not cross 0, indicating that all effects were significant, GenAI, generative artificial intelligence; TI, teaching innovation; LM, learning motivation; PAD, professional autonomy development.

### Moderated conditional process analysis

The moderated conditional process model showed that the direct association between GenAI acceptance and PAD remained stable (effect = 0.332, 95% CI [0.272, 0.392]), while the indirect associations through teaching innovation and learning motivation varied by technology anxiety. Specifically (Table 6), technology anxiety negatively moderated the associations between GenAI acceptance and both intermediate variables. The interaction coefficients were −0.240 for teaching innovation and −0.179 for learning motivation (both p < 0.001), indicating that higher technology anxiety attenuated the positive associations between GenAI acceptance and teaching innovation and learning motivation.

**Table 6:** Moderated conditional process analysis.

Model	Dependent variable	Predictor variable	Overall fit indices			Regression coefficients	
			R	R^2^	f	Effect	t
1	TI	GenAI	0.568	0.322	160.893	0.98	12.448^***^
		TA				0.666	6.065^***^
		GenAI^*^TA				−0.24	−8.198^***^
2	LM	GenAI	0.659	0.435	195.114	0.846	10.833^***^
		TI				0.199	6.859^***^
		TA				0.481	4.655^***^
		GenAI^*^TA				−0.179	−6.414^***^
3	PAD	GenAI	0.639	0.409	233.995	0.332	10.88^***^
		TI				0.232	8.166^***^
		LM				0.202	6.763^***^

^***^p < 0.001, GenAI, generative artificial intelligence; TI, teaching innovation; LM, learning motivation; PAD, professional autonomy development; TA, technology anxiety.

Because the models included interaction terms, the lower-order coefficient of technology anxiety should not be interpreted as a zero-order association. Substantive interpretation therefore focused on the GenAI acceptance × technology anxiety interaction, simple slope estimates, and conditional indirect effects.

**Figure 3:**
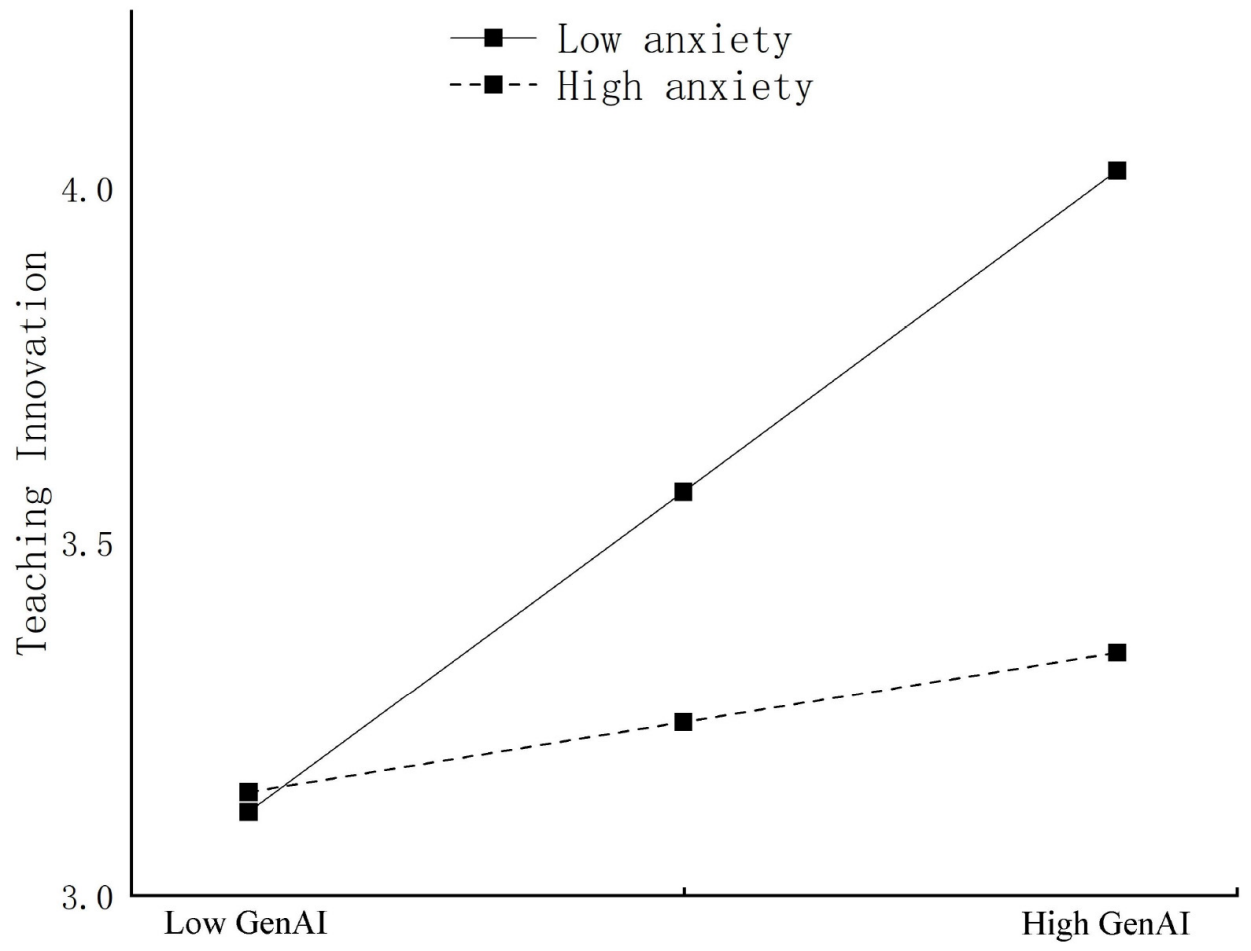
Moderating association of technology anxiety between GenAI acceptance and teaching innovation.

**Figure 4:**
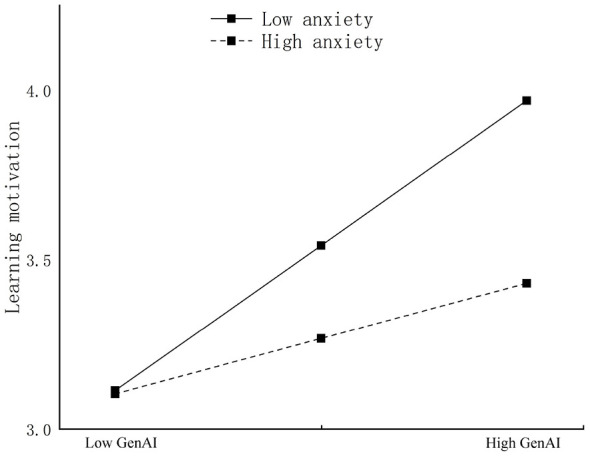
Moderating association of technology anxiety between GenAI acceptance and learning motivation.

Simple slope analyses further supported the moderation effect. At one standard deviation below the mean of technology anxiety (low anxiety; TA = 1.678 = M - 1 SD), the association between GenAI acceptance and teaching innovation was strong and significant (effect = 0.577, *p* < 0.001, 95% CI [0.503, 0.651]); at one standard deviation above the mean (high anxiety; TA = 3.556 = M + 1 SD), this association remained significant but was substantially weaker (effect = 0.126, *p* = 0.003, 95% CI [0.042, 0.210]) (Figure 3). Similarly, the association between GenAI acceptance and learning motivation decreased from 0.545 at low technology anxiety (TA = 1.678; *p* < 0.001, 95% CI [0.469, 0.621]) to 0.208 at high technology anxiety (TA = 3.556; *p* < 0.001, 95% CI [0.131, 0.286]) (Figure 4).

Technology anxiety also shaped the three indirect associations from GenAI acceptance to PAD (Table 7). Under low technology anxiety, the independent indirect associations through teaching innovation and learning motivation were 0.134 (95% CI [0.096, 0.173]) and 0.110 (95% CI [0.075, 0.149]), respectively, and the serial indirect association was 0.023 (95% CI [0.014, 0.034]). Under high technology anxiety, the corresponding association sizes declined by 78.4%, 61.8%, and 78.3%, respectively. The attenuation was strongest for the teaching innovation association, which decreased to 0.029 (95% CI [0.008, 0.051]). The serial indirect association remained statistically significant but was very small (effect = 0.005, 95% CI [0.001, 0.010]). These findings suggest that technology anxiety is particularly associated with reduced innovation attempts, whereas the learning motivation association appears relatively more resilient.

**Table 7:** Indirect association effect sizes under different levels of technology anxiety.

Pathway	Technology anxiety level	Effect	Boot SE	95% confidence interval	
				LLCI	ULCI
Direct effect		0.332	0.031	0.272	0.392
GenAI ->TI-> PAD	Low anxiety	0.134	0.019	0.096	0.173
	High anxiety	0.029	0.011	0.008	0.051
GenAI ->LM-> PAD	Low anxiety	0.11	0.019	0.075	0.149
	High anxiety	0.042	0.010	0.023	0.065
GenAI ->TI-> LM ->PAD	Low anxiety	0.023	0.005	0.014	0.034
	High anxiety	0.005	0.002	0.001	0.01

The confidence interval of the pathway did not cross 0, indicating that all effects were significant, GenAI, generative artificial intelligence; TI, teaching innovation; LM, learning motivation; PAD, teacher professional autonomy development; TA, technology anxiety.

## Discussion

The findings indicate that GenAI acceptance was positively associated with PAD among Chinese university physical education teachers. Teaching innovation and learning motivation showed significant independent indirect associations and a smaller serial indirect association. Technology anxiety weakened the associations between GenAI acceptance and both intermediate variables. Taken together, the results suggest that GenAI acceptance is associated with PAD partly through innovation-related and motivational processes, while the strength of these associations varies with teachers' technology anxiety.

### The indirect role of teaching innovation

GenAI acceptance was positively associated with teaching innovation (β = 0.477, *p* < 0.001), which was subsequently associated with PAD (β = 0.236, *p* < 0.001). The indirect association through teaching innovation was 0.113 (95% CI [0.084, 0.146]), accounting for 20.07% of the total association. Teaching innovation was therefore the strongest of the three indirect pathways examined.

One possible interpretation is that teachers who regard GenAI tools as useful and manageable are more willing to experiment with their instructional practices. In physical education, such experimentation may include generating differentiated instructional materials, developing alternative lesson plans, interpreting student performance information, or providing more individualized feedback (Abbes et al., 2024; Hashim et al., 2023). These activities may expand teachers' perceived range of pedagogical choices and support reflective instructional decision-making. Nevertheless, GenAI acceptance should not be equated with innovation itself. Teachers must evaluate, adapt, and integrate AI-generated materials into specific instructional environments before technological acceptance can become meaningful pedagogical change (Gabriel, 2024).

### The indirect role of learning motivation

Learning motivation also showed an independent indirect association between GenAI acceptance and PAD. GenAI acceptance was positively associated with learning motivation (β = 0.425, *p* < 0.001), and learning motivation was positively associated with PAD (β = 0.208, *p* < 0.001). The corresponding indirect association was 0.089 (95% CI [0.059, 0.122]), accounting for 15.81% of the total association.

This finding can be interpreted through self-determination theory, which proposes that autonomy, competence, and relatedness support internalized motivation and sustained engagement in learning (Deci and Ryan, 2000; Ryan and Deci, 2020). GenAI tools may make professional learning resources more accessible, provide rapid feedback, and allow teachers to explore instructional problems at their own pace (Boguslawski et al., 2024; Chiu, 2024; Du and Alm, 2024). When teachers perceive these functions as useful, they may feel more capable of addressing professional challenges and become more willing to engage in continued learning (Hazzan-Bishara et al., 2025).

However, the learning motivation measure used in this study encompasses cognitive learning, career-development, and social-recognition motives. It should therefore not be interpreted as a measure of purely intrinsic motivation. The observed pathway may reflect a combination of interest in acquiring knowledge, perceived career value, and expectations of professional recognition.

### The serial indirect association of teaching innovation and learning motivation

In addition to the two independent indirect associations, the serial path through teaching innovation and learning motivation was statistically significant. The serial indirect association was 0.029 (95% CI [0.018, 0.041]), accounting for 5.15% of the total association. Although this association was smaller than either independent indirect association, it is consistent with the possibility that teaching innovation and learning motivation operate as connected rather than isolated professional processes. The temporal ordering of these variables, however, cannot be established by the main-survey design.

Within the hypothesized ordering, GenAI acceptance was associated with greater teaching innovation, which in turn was associated with stronger learning motivation. Successful experimentation may provide mastery experiences, make the professional value of further learning more visible, and reveal areas in which additional knowledge is needed (An et al., 2022; Collie and Martin, 2024; Hwang and Wu, 2025; Yu et al., 2021). These interpretations remain provisional because the variables were not measured at separate longitudinal time points.

The relatively small serial association should be regarded as a complementary model-based pattern rather than the dominant mechanism or a demonstrated temporal sequence. Teachers may become motivated to learn without first changing their teaching practices, while teaching innovation may be associated with PAD without necessarily producing sustained learning motivation. Productive engagement with GenAI tools should also be distinguished from habitual dependence. Du et al. (2026) identified factors associated with teachers' dependence on GenAI within the I-PACE framework, underscoring the importance of reflective control and independent professional judgment.

### The moderating role of technology anxiety

Technology anxiety weakened the associations between GenAI acceptance and teaching innovation (β = −0.240, *p* < 0.001) and between GenAI acceptance and learning motivation (β = −0.179, *p* < 0.001). The serial indirect association decreased from 0.023 (95% CI [0.014, 0.034]) at low technology anxiety to 0.005 (95% CI [0.001, 0.010]) at high technology anxiety. Technology anxiety therefore appears to constrain the conversion of favorable GenAI evaluations into innovation and professional learning (Bae et al., 2024; Wang et al., 2024).

The stronger moderation of the teaching innovation pathway is theoretically plausible because innovation requires experimentation under uncertainty. Teachers experiencing technology anxiety may avoid unfamiliar functions, devote cognitive resources to concerns about errors, or lack confidence in evaluating AI-generated outputs (Caporusso, 2023; Pettersson, 2018). Learning motivation may be somewhat less vulnerable because it can also be supported by career goals, perceived usefulness, and expectations of professional recognition. These responses may be amplified in contexts where fear of failure and reputational concerns are salient (Wennberg et al., 2013).

Recent evidence indicates that teachers' responses to ChatGPT involve interrelated cognitive evaluations, affective reactions, self-efficacy, and facilitating conditions (Wang et al., 2026). The present results extend this perspective by suggesting that technology anxiety may operate as a boundary condition that weakens the association of GenAI acceptance with innovation and professional learning, rather than functioning only as a direct antecedent of acceptance.

Reducing anxiety should nevertheless not mean encouraging uncritical or unrestricted use. Du et al. (2026) showed that cognitive and affective factors within the I-PACE framework were associated with teachers' dependence on GenAI. Institutions should therefore balance psychological and technical support for hesitant users with guidance that preserves active cognitive engagement and professional judgment among intensive users.

### Limitations and future directions

Several limitations should be considered. First, the preliminary pilot survey supported questionnaire refinement, but the formal conditional process model was estimated from the main questionnaire survey. The main variables in the indirect model were therefore not measured at separate longitudinal time points, and the study cannot establish causal relationships or temporal sequencing. Future studies should use multiple measurement waves, cross-lagged designs, experiments, or professional-development interventions to examine temporal ordering more rigorously.

Second, all constructs were measured using self-reports, and the mediation analysis used composite scores rather than latent variables. Although the five-factor measurement model, HTMT results, and common-method assessments supported construct distinctiveness, measurement error and residual common-method variance cannot be excluded. Future studies could incorporate classroom observations, GenAI usage logs, instructional materials, and evaluations from students or colleagues.

Third, some measures were translated, contextually adapted, rescaled, or shortened. Du (2024) emphasizes that educational scale adaptation requires transparent documentation, content assessment, pilot testing, statistical validation, and, where possible, cross-validation. The present study included a preliminary pilot survey, translation and back-translation, expert review, reliability assessment, factor analysis, and additional measurement-invariance tests across gender and teaching-experience groups. However, item refinement, invariance testing, and the main model analyses relied on the same main-survey dataset. The psychometric findings should therefore be interpreted as evidence within the present sample rather than independent external validation. Future research should cross-validate the adapted measures in independent and more diverse teacher samples. In particular, future research should examine measurement invariance across additional grouping variables, such as institutional type, academic rank, regional context, digital literacy level, and disciplinary background.

Finally, the model emphasized potential professional-development benefits associated with GenAI acceptance but did not directly assess overreliance, cognitive offloading, professional deskilling, or threats to professional identity. Evidence that teachers may develop dependence on GenAI demonstrates the need to examine beneficial and adverse patterns simultaneously. Future models should investigate whether reflective use, AI literacy, or institutional governance conditions the association between GenAI acceptance and PAD.

### Practical implications

Universities should adopt a differentiated approach to GenAI-supported teacher development. Teachers with high technology anxiety may benefit from guided practice, peer support, low-risk experimentation, and reliable technical assistance (Collie and Martin, 2024; Hwang and Wu, 2025). Training should also extend beyond operational competence. Teachers need critical AI literacy to evaluate the accuracy, pedagogical suitability, ethical implications, and limitations of AI-generated outputs. Institutions should require human oversight in instructional and assessment decisions and encourage teachers to document why and how GenAI tools are used.

Finally, professional-development programs should help teachers distinguish productive assistance from habitual reliance. Reflective prompts, periodic evaluation of AI-supported decisions, and tasks requiring independent instructional design may help preserve professional judgment. GenAI should complement rather than replace teachers' cognitive engagement, creativity, and professional autonomy.

## Conclusion

This study found that GenAI acceptance was positively associated with PAD among university physical education teachers. Teaching innovation and learning motivation showed significant indirect associations, and technology anxiety weakened the associations between GenAI acceptance and both intermediate variables, particularly teaching innovation. Because teaching innovation, learning motivation, and PAD were measured in the same main survey, these findings should be interpreted as regression-based conditional process associations rather than evidence of causal, cross-lagged, or developmental effects. The results highlight the importance of technical support, critical AI literacy, psychological support, and clear ethical governance while preserving teacher agency and professional judgment.

## Data Availability

The raw data supporting the conclusions of this article will be made available by the authors, without undue reservation.
